# Evaluating Consensus Treatment Plans for Chronic Nonbacterial Osteomyelitis Using Real-World Data

**DOI:** 10.3390/children13101410

**Published:** 2026-10-15

**Authors:** Nebahat Zeynep Özaslan, Eray Tunce, Şerife Tuncez, Bengisu Menentoğlu, Betül Sözeri, Semanur Özdel, Nuray Aktay Ayaz, Serkan Türkuçar, Selçuk Yüksel, Mukaddes Kalyoncu, Sıla Atamyıldız Uçar, Semra Ayduran, Şeyma Türkmen, Fırat Gündoğmuş, Merve Özen Balcı, Kübra Öztürk, Nihal Karacayır, Sevcan Bakkaloğlu, Gökçe Sumer, Sara Şebnem Kılıç, Mustafa Çakan, Kadir Ulu, Hatice Adıgüzel Dündar, Ferhat Demir, Burcu Bozkaya, Hande Ilgaz Tüzen, Belde Kasap Demir, Figen Çakmak, Özge Altuğ Gücenmez, Fatma Gül Demirkan, Nihal Şahin, Hafize Emine Sönmez

**Affiliations:** 1 Department of Pediatric Rheumatology, Faculty of Medicine, Kocaeli University, 41380 Kocaeli, Türkiye; nihal.sahin@kocaeli.edu.tr (N.Ş.); eminesonmez@gmail.com (H.E.S.); 2 Department of Pediatric Rheumatology, Ümraniye Training and Research Hospital, University of Health Sciences, 34764 Istanbul, Türkiye; eraytunce@gmail.com (E.T.); drbetulsozeri@gmail.com (B.S.); satamyildiz@gmail.com (S.A.U.); drpieni@hotmail.com (Ş.T.); 3 Department of Pediatric Rheumatology, Etlik City Hospital, 06170 Ankara, Türkiye; drserifetuncez@gmail.com (Ş.T.); semanurozdel@gmail.com (S.Ö.); 4 Department of Pediatric Rheumatology, Faculty of Medicine, İstanbul University, 34452 Istanbul, Türkiye; bengisusanisoglu@gmail.com (B.M.); nurayaktay@gmail.com (N.A.A.); 5 Department of Pediatric Rheumatology, Faculty of Medicine, Pamukkale University, 20160 Pamukkale, Türkiye; serkan_turkucar@hotmail.com (S.T.); semraayduran@yahoo.com (S.A.); 6 Department of Pediatric Rheumatology, Faculty of Medicine, Çanakkale Onsekiz Mart University, 17100 Çanakkale, Türkiye; selcukyuksel@yahoo.com; 7 Department of Pediatric Rheumatology, Faculty of Medicine, Karadeniz Technical University, 61080 Trabzon, Türkiye; mukaddeskalyoncu@gmail.com (M.K.); firat.gundogmus@ktu.edu.tr (F.G.); 8 Department of Pediatric Rheumatology, Istanbul Medeniyet University, Göztepe Prof. Dr. Süleyman Yalçın City Hospital, 34700 Istanbul, Türkiye; merveozen58@gmail.com (M.Ö.B.); ozturk1209@gmail.com (K.Ö.); 9 Department of Pediatric Rheumatology, Faculty of Medicine, Gazi University, 06560 Ankara, Türkiye; nihal_karacayir@hotmail.com (N.K.); sevcan@gazi.edu.tr (S.B.); 10 Department of Pediatric Rheumatology, Faculty of Medicine, Bursa Uludag University, 16059 Bursa, Türkiye; gokcesumer.90@gmail.com (G.S.); sebnemkl@uludag.edu.tr (S.Ş.K.); 11 Department of Pediatric Rheumatology, Zeynep Kamil Women and Children’s Diseases Training and Research Hospital, 34668 Istanbul, Türkiye; mustafacakan@hotmail.com; 12 Department of Pediatric Rheumatology, Sancaktepe Prof. Dr. Ilhan Varank Training and Research Hospital, University of Health Sciences, 34668 Istanbul, Türkiye; drkadirulu@gmail.com; 13 Department of Pediatric Rheumatology, Dr. Behçet Uz Pediatric Diseases and Surgery Training and Research Hospital, 35210 Izmir, Türkiye; haticeadiguzel@hotmail.com (H.A.D.); droaltug@hotmail.com (Ö.A.G.); 14 Department of Pediatric Rheumatology, Acıbadem Ataşehir Hospital, 34642 Istanbul, Türkiye; drferhatdemir@gmail.com; 15 Department of Pediatric Rheumatology, Samsun Training and Research Hospital, 55090 Samsun, Türkiye; bozkayaburcu@hotmail.com; 16 Department of Pediatric Rheumatology and Nephrology, İzmir Katip Çelebi University, 35620 Izmir, Türkiye; hande.ilgaz@deu.edu.tr (H.I.T.); beldekasap@gmail.com (B.K.D.); 17 Department of Pediatric Rheumatology, Başakşehir Çam and Sakura City Hospital, 34480 Istanbul, Türkiye; figen.cakmak@istanbul.edu.tr; 18 Department of Pediatric Rheumatology, Kanuni Sultan Süleyman Training and Research Hospital, 34303 Istanbul, Türkiye; fatmagy@gmail.com

**Keywords:** chronic nonbacterial osteomyelitis, consensus treatment plan, clinical disease activity score

## Abstract

**Highlights:**

**What are the main findings?**

• Tumor necrosis factor (TNF) inhibitors were the most frequently used treatment strategy in patients with more severe chronic nonbacterial osteomyelitis (CNO), particularly those with spinal involvement or a higher magnetic resonance imaging (MRI) lesion burden, and achieved the highest remission rate.
• The Chronic Nonbacterial Osteomyelitis Clinical Disease Activity Score (CNO-CDAS) markedly decreased during follow-up, with most patients achieving either medicated or drug-free remission at the final visit.

**What are the implications of the main findings?**

• In this multicenter retrospective cohort of 171 children with chronic nonbacterial osteomyelitis followed for a median of 36 months, disease activity significantly improved, and 91.2% achieved remission at the last visit.
• Childhood Arthritis and Rheumatology Research Alliance (CARRA) consensus treatment plans provide a useful framework for real-world treatment decisions in pediatric CNO, particularly for patients with refractory or severe disease.
• CNO-CDAS appears to be a practical clinical tool for monitoring disease activity and treatment response and may be integrated into routine clinical practice.

**Abstract:**

**Introduction:** Chronic nonbacterial osteomyelitis (CNO) is a rare autoinflammatory bone disorder. To standardize management, the Childhood Arthritis and Rheumatology Research Alliance (CARRA) developed three consensus treatment plans (CTPs) (1: methotrexate or sulfasalazine [conventional disease-modifying antirheumatic drugs; DMARDs], 2: tumor necrosis factor (TNF)-alpha inhibitors (TNFi), and 3: bisphosphonates). The Clinical Disease Activity Score (CNO CDAS) was introduced to assess disease activity without relying solely on imaging. **Objective:** The objectives were to evaluate clinicians’ tendencies in applying CNO-CTPs and to assess treatment responses using CNO CDAS in real-world practice, including therapeutic strategies and outcomes at final follow-up. **Methods:** A retrospective cohort study of 171 pediatric patients with CNO from 18 centers was conducted. Disease activity was assessed using CDAS at diagnosis, at treatment modification, and at the last visit. This score incorporates clinically active lesions, patient pain, and global assessment. Treatments included NSAIDs, DMARDs, TNFi, and pamidronate. **Results:** Median age was 10 years at onset and 14 years at last follow-up, with a median follow-up of 36 months. The most frequent symptoms were bone pain (95.3%), activity limitation (67.8%), and fatigue (35.1%). All patients received NSAIDs; methotrexate (85.4%) and TNFi (54.4%) were commonly used. TNF inhibitors were the most used CTP, especially in patients with spinal involvement or higher MRI lesion counts, and they achieved the highest remission rate (40.4%). Of the 89 (52.0%) patients who changed treatment, 93.2% switched from DMARDs to TNFi. At the final follow-up, 62% were in medicated remission, 29.2% were in unmedicated remission, and 8.8% had active disease. CDAS scores significantly improved over time (*p* < 0.05). **Conclusions:** CARRA CTPs are effective in clinical practice, with anti-TNF therapy yielding the highest remission rates and marked improvement in CDAS.

## 1. Introduction

Chronic nonbacterial osteomyelitis (CNO) is a rare autoinflammatory bone disorder predominantly affecting children and adolescents. Its clinical spectrum ranges from mild unifocal involvement to severe, chronic, or recurrent multifocal bone inflammation. Delayed diagnosis or inadequate treatment may lead to significant complications, including vertebral compression fractures, kyphotic deformities, and limb length discrepancies [1,2,3,4,5,6]. Despite its serious consequences, treatment for CNO is often administered empirically, guided by personal experience, expert opinion, and retrospective observational data [7,8,9,10,11,12,13,14]. Given the substantial variability in clinical management among pediatric rheumatologists and the limited evidence supporting the efficacy of treatment options in CNO, the Childhood Arthritis and Rheumatology Research Alliance (CARRA) CNO Working Group developed Consensus Treatment Plans (CTPs) to reduce variation within treatment regimens and facilitate comparative effectiveness research in children with disease refractory to non-steroidal anti-inflammatory drugs (NSAIDs) and/or active spinal involvement. These CTPs were not intended to serve as clinical practice guidelines or to establish the superiority of any treatment approach. These CTPs comprise three treatment plans: (1) methotrexate (MTX) or sulfasalazine (SSZ), (2) tumor necrosis factor (TNF)-alpha inhibitors (TNFi), which may be combined with MTX, and (3) bisphosphonates [1].

Assessing disease activity in patients with CNO is challenging due to the absence of standardized evaluation tools and sensitive laboratory markers. The Chronic Nonbacterial Osteomyelitis International Registry (CHOIR), an international, observational, and longitudinal cohort study, was established in 2018 by the CARRA CNO Working Group. This study enrolled children with NSAID-refractory disease, lesions associated with a high risk of permanent skeletal damage, or both. Follow-up assessments were performed at 3, 6, 9, 12, 18, and 24 months, which contributed to the development of the Clinical Disease Activity Score (CDAS) for CNO. The CNO CDAS is calculated by summing patient-reported pain, the patient/parent global assessment of disease activity, and the total number of clinically active CNO lesions [1,15].

This study aimed to describe real-world treatment patterns in relation to the therapeutic approaches included in the CARRA CTPs, assess treatment responses using the CDAS, and analyze treatment trajectories and outcomes at the final follow-up visit in pediatric patients with CNO who were refractory to NSAIDs and/or had active spinal lesions.

## 2. Materials and Methods

This retrospective, multicenter, longitudinal observational study was conducted by members of the Pediatric Rheumatology Academy (PeRA) research group. A total of 171 patients under the age of 18 who were diagnosed with CNO and who showed an inadequate response to NSAID treatment and/or had active spinal lesions were included in the study. The diagnosis of CNO was based on the criteria established by Jansson et al. [16]. The inclusion criteria for the study were as follows: diagnosis made before the age of 18; presence of bone edema on magnetic resonance imaging (MRI) using STIR or T2 fat-suppressed sequences; lack of response to at least 4 weeks of NSAID use and/or presence of active spinal lesions (active disease regardless of NSAID treatment); and bone biopsy performed to exclude infection or malignancy (biopsy was not required in cases with lesions consistent with a typical CNO distribution).

Patients older than 18 years at the time of diagnosis and those with any concomitant systemic disease or another rheumatic disease were excluded. None of the included patients had a concomitant diagnosis of juvenile idiopathic arthritis (JIA) or inflammatory bowel disease, and patients with arthritis were not treated as having overlapping JIA.

Each of the 18 participating centers reviewed its own medical records to identify eligible patients with CNO. Patients were included if they met the Jansson criteria, had no comorbid diseases, attended regular follow-up, and had complete data for the study variables. Each center contributed data only for patients meeting these eligibility criteria. The total number of records screened and patients excluded, as well as the reasons for exclusion, were not collected centrally.

Patients’ demographic data, laboratory findings, imaging results, treatments, and treatment responses were retrospectively reviewed using the medical records. Based on the use of the proposed CTPs, patients were classified into three groups: (1) conventional synthetic disease-modifying antirheumatic drugs (csDMARDs), such as MTX and SSZ; (2) TNFi, with or without MTX; and (3) bisphosphonates, including pamidronate and zoledronic acid. Limited use of NSAIDs and glucocorticoids was permitted across all treatment regimens [1]. Disease activity was assessed at diagnosis, at the time of treatment modification, and at the final visit using the CNO CDAS, which is calculated based on the number of clinically active lesions, patient-reported pain assessment, and global disease activity parameters. CNO-CDAS was calculated retrospectively for all patients by the pediatric rheumatologists at each participating center using clinical information documented during routine care. Responses to the CTPs—comprising DMARDs, biologic agents (anti-TNF therapies), and pamidronate—were analyzed.

Clinical assessments were performed during routine care. For this retrospective study, pediatric rheumatologists at each participating center reviewed the medical records and categorized treatment responses as remission, partial response, or no response using the following definitions. Clinical remission was defined as normalization of inflammatory markers (erythrocyte sedimentation rate [ESR] < 20 mm/h and C-reactive protein [CRP] < 5 mg/L), absence of inflammatory pain, and absence of clinically active lesions. Radiological remission was defined as resolution of bone marrow edema and absence of new lesions on MRI. Partial response was defined as improvement in clinical signs and symptoms compared with pretreatment status without meeting the criteria for clinical remission. No response was defined as the absence of improvement in clinical signs and symptoms. Patients with persistent inflammatory pain despite resolution of MRI lesions were considered to have achieved radiological remission but not clinical remission and were classified as having a partial response or no response according to whether clinical improvement had occurred. These definitions were informed by the CARRA consensus criteria for treatment failure in CNO [1]. Arthritis was identified based on clinical assessment; imaging confirmation was not required.

On MRI, lesions were described as areas of abnormal hyperintensity on short tau inversion recovery (STIR) sequences, abnormal hypointensity on T1-weighted images, and/or regions showing contrast enhancement. Active spinal lesions were defined based on MRI findings, including bone marrow edema (hyperintensity), abnormal contrast enhancement, loss of vertebral height, vertebral compression, and bone expansion (hyperostosis) [17]. Radiologists with expertise in musculoskeletal MRI evaluated all imaging studies.

### Statistical Analysis

A database was created in SPSS (version 32) for statistical analysis. The normality of variable distributions was evaluated using visual methods (histograms and probability plots) and analytical tests (Kolmogorov–Smirnov/Shapiro–Wilk tests). Descriptive statistics were used to summarize demographic and clinical characteristics. The median and interquartile range (IQR) were calculated for continuous variables; frequencies and percentages were reported for categorical variables. To assess significant differences in baseline patient characteristics among CTPs, either the Mann–Whitney U test or Fisher’s exact test was applied. The Friedman test was conducted to compare related samples. Participants with missing data or who were lost to follow-up were excluded from the respective analyses.

## 3. Results

### 3.1. Baseline Characteristics of Participants

A total of 171 patients diagnosed with CNO who were unresponsive to NSAIDs and/or had spinal lesions were included in the study. Of these, 68 (39.8%) were female and 103 (60.2%) were male. The median age at symptom onset was 10 years (IQR: 8–12), the median age at diagnosis was 11 years (IQR: 8.2–13), and the median age at the last visit was 14 years (IQR: 12–16). The median follow-up duration was 36 months (IQR: 18–52.7). A family history of rheumatic disease was reported in 25 patients (14.6%): 10 (5.8%) had chronic arthritis, 5 (2.9%) had CNO, 5 (2.9%) had familial Mediterranean fever, and 5 (2.9%) had spondyloarthritis.

At clinical presentation, pain was most commonly localized to the lower extremities in 113 patients (66.1%), followed by the pelvis in 58 patients (33.9%), the upper extremities in 43 patients (25.1%), the vertebrae in 27 patients (15.8%), the clavicle in 23 patients (13.4%), the ribs in 5 patients (2.9%), and the skull bones in 3 patients (1.8%). Arthritis was observed in 82 patients (47.9%). Among those with arthritis, the most frequently affected joints were the foot and ankle in 32 patients (39.0%), the knee and sacroiliac joints in 20 patients each (24.4% each), the hips in 12 patients (14.6%), and the hand/wrist, elbow, and temporomandibular joint in 3 patients each (3.7%).

At the time of diagnosis, X-ray imaging was performed in 92 patients (53.8%), and pathological findings were identified in 20 (11.7%). Furthermore, 139 patients (81.3%) underwent regional MRI; of these, 39 (22.8%) also underwent whole-body MRI. The remaining 32 patients (18.7%) underwent only a whole-body MRI. The median number of lesions detected on the whole-body MRI was 6 (IQR: 2–11). MRI findings included bone marrow edema in 98 patients (57.3%), soft tissue edema in 42 patients (24.6%), osteitis in 25 patients (14.6%), synovitis in 16 patients (9.4%), sclerosis in 12 patients (7.1%), and osteopenia or osteoporosis in 2 patients (1.2%). Sacroiliitis was detected in 59 patients (34.5%) and spinal involvement in 35 patients (20.5%). Bone mineral density (BMD) was assessed in 17 patients (9.9%), and all results were reported as normal. Bone biopsy was performed in 49 patients (28.7%), revealing a mixed inflammatory infiltrate with varying degrees of sclerosis and fibrosis. Additional imaging modalities were bone scintigraphy (43 patients, 25.1%) and PET-CT (4 patients, 2.3%).

### 3.2. Treatments and Outcomes

All 171 patients (100%) had previously received NSAIDs. Subsequently, 146 (85.4%) received MTX; 41 (24.0%) received SSZ; 93 (54.4%) received anti-TNF agents; and 14 (8.1%) received pamidronate. Among those treated with anti-TNF agents, 48 (51.6%) received etanercept, 38 (40.9%) received adalimumab, and 7 (7.5%) received infliximab. Thirty-seven patients (39.8%) received concomitant methotrexate, while 56 (60.2%) received anti-TNF monotherapy. No patients developed psoriasis during anti-TNF treatment. None of the patients received corticosteroids during the study period.

**Figure 1:**
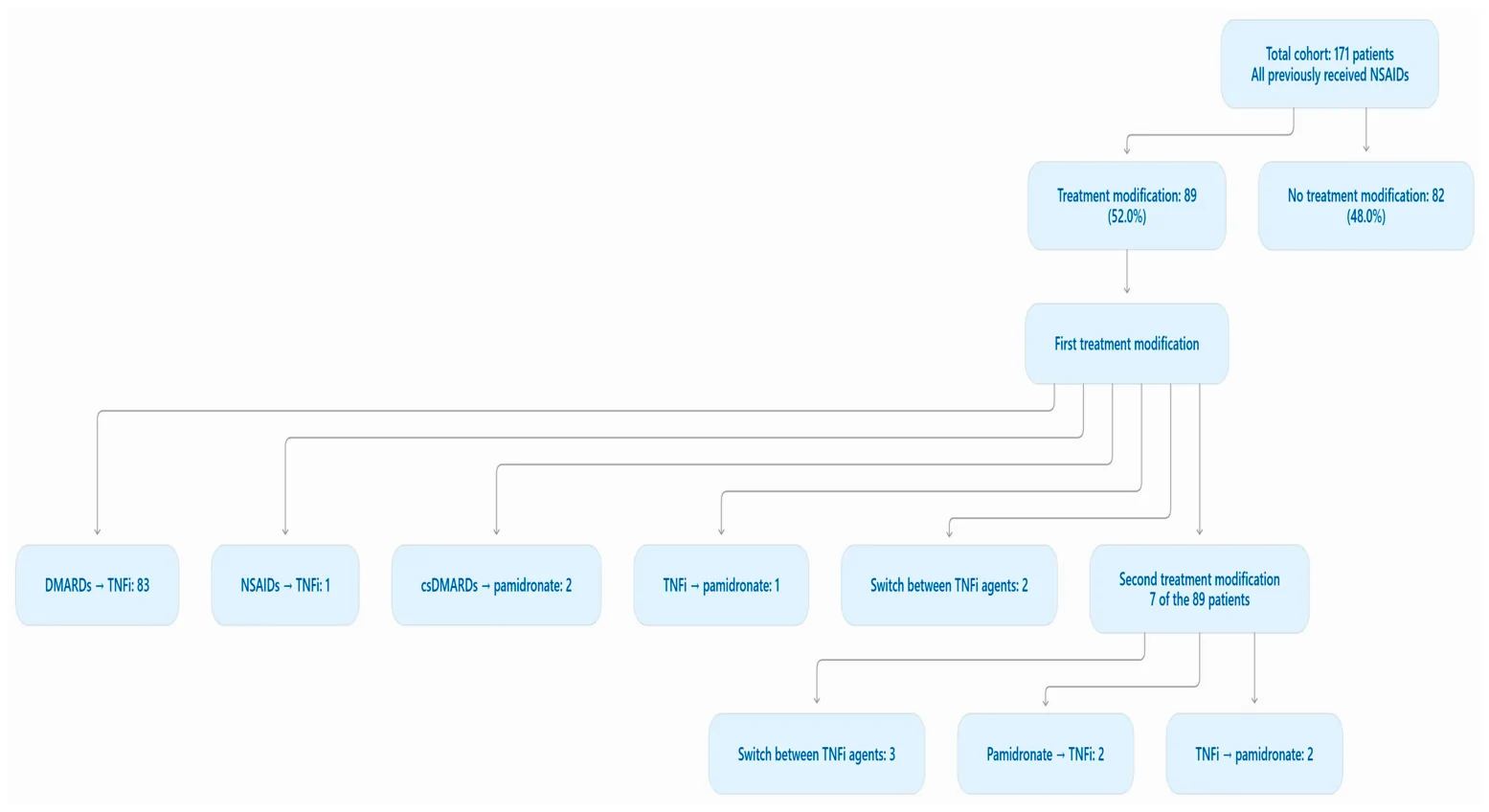
**Treatment modifications in 171 pediatric patients with chronic nonbacterial osteomyelitis.** Eighty-nine patients underwent a first treatment modification; seven of these subsequently underwent a second modification. Individual trajectories linking the first and second modifications are not shown. DMARDs, disease-modifying antirheumatic drugs; csDMARDs, conventional synthetic DMARDs; TNFi, tumor necrosis factor inhibitors; NSAIDs, non-steroidal anti-inflammatory drugs.

Treatment modifications were made for a total of 89 patients. Of these, 83 patients transitioned from DMARDs to TNFi; 1 patient switched from NSAIDs to TNFi; 2 patients switched from csDMARDs to pamidronate; 1 patient switched from TNFi to pamidronate; and 2 patients switched between different TNFi agents (Figure 1). Among the 89 patients who underwent treatment modification, the median time from initiation of the initial treatment to the first modification was 3 months (3–12). Additionally, seven patients underwent a second treatment modification: three underwent a TNFi switch, two transitioned from pamidronate to TNFi, and two transitioned from TNFi to pamidronate. Clinical and laboratory findings, imaging results, and disease activity scores were evaluated at diagnosis, at treatment modification, and at the last follow-up visit. Data at diagnosis and the last visit were available for all 171 patients, whereas treatment modification data were available only for the 89 patients who underwent a treatment change. Bone pain was the most common clinical symptom at all assessment time points. CRP and ESR levels at these time points are summarized descriptively in Table 1. Details of treatment durations and responses are presented in Table 2.

In the treatment modifications group (89 patients), 13 (14.6%) underwent X-rays; pathological findings were identified in 3 cases (3.4%). Regional MRI was performed in 25 patients (28.1%) and whole-body MRI was performed in 11 patients (12.4%). Sacroiliitis, spinal involvement, and arthritis were detected in 19 (21.3%), 8 (9.0%), and 20 (22.5%) patients, respectively.

At the final visit, regional MRI was performed in 22 patients (12.9%), and whole-body MRI was performed in 20 patients (11.7%). These imaging studies revealed sacroiliitis in 6 patients (3.5%), spinal involvement in 1 patient (0.6%), and arthritis in 5 patients (2.9%). The most affected sites were the metaphyseal regions of the lower extremities.

At the time of diagnosis, the median CNO-CDAS score was 15 (IQR: 13–18), and this value remained unchanged at the time of treatment modification. By the final visit, a significant reduction was observed, with the median CDAS score decreasing to 0 (IQR: 0–5). The CDAS scores at the final visit were significantly lower than both the scores at diagnosis and those at the time of treatment modification. The reported lesion counts represent clinically active lesions and do not necessarily reflect the total lesion burden on MRI. In contrast, no statistically significant difference was found between the scores at diagnosis and at the time of treatment modification (Table 3). At final follow-up, 106 patients (62%) achieved drug-induced remission, 50 (29.2%) achieved drug-free remission, and 15 (8.8%) continued to have active disease. No patients developed inflammatory bowel disease (IBD) during the study period. Enthesitis was present in 19 patients (11.1%) at diagnosis and 5 patients (2.9%) at the final visit, while sacroiliitis was detected in 59 patients (34.5%) at diagnosis and 6 patients (3.5%) at the final visit.

A comparison was made between treatment groups, specifically patients using csDMARDs alone and those whose treatment was changed because of insufficient response to csDMARDs. However, since there were only 2 patients who switched from csDMARDs to pamidronate, 1 patient who switched from TNFi to pamidronate, and 2 patients who switched between TNFi treatments, given the sample sizes in these groups, a binary comparison was conducted only between patients using DMARDs and those who switched to TNFi (Table 4). Disease duration was longer, and both the total lesion count on MRI and the proportion of patients with active spinal lesions was higher in the TNFi group. The comparison of CNO CDAS scores among treatment groups is shown in Table 5.

## 4. Discussion

This multicenter, retrospective study aimed to evaluate disease activity in pediatric CNO patients using the CDAS, incorporating clinical, imaging, and treatment data; to assess the efficacy of consensus treatment plans (CTPs); to examine clinicians’ tendencies in the use of CTPs; and to analyze treatment responses, therapeutic processes, and outcomes at the final visit based on real-world data in patients with disease refractory to NSAIDs and/or active spinal lesions. TNF inhibitors (TNFi) were the preferred CTP regimen, particularly among patients with spinal involvement or a higher number of MRI lesions and yielded the highest remission rate (40.4%) among the 171 patients followed for a median of 36 months.

Consensus treatment plans are standardized strategies developed by experts to guide the management of diseases, especially in areas with limited high-quality evidence. In pediatric rheumatology, CARRA has led the development of CTPs for conditions such as CNO, aiming to reduce practice variability and to facilitate research. In clinical settings, CTPs ensure consistency in care, support shared decision-making, and contribute to real-world data for evidence-based guidelines. Advantages include improved comparability of outcomes and facilitation of multicenter studies. However, CTPs may lack flexibility, struggle to keep pace with new treatments, and depend on clinician adherence and infrastructure. Unmet needs include CTPs for rare disease subtypes, improved integration of patient-reported outcomes, and adaptation for use across diverse healthcare systems. Therefore, it is important to understand the extent to which clinicians implement these CTPs in real-world clinical practice and how effective they are in achieving treatment responses. This study makes a significant contribution to the literature by evaluating, for the first time, the clinical application of CDAS in a large, multicenter sample and by analyzing treatment responses using CTPs.

In a survey-based study of clinicians managing patients with CNO, nearly all respondents (95%) reported prescribing NSAIDs as the initial treatment modality. The most common second-line agents following NSAID failure were MTX (67%), TNFi (65%), and bisphosphonates (46%) [18]. In our cohort, 100% of patients received NSAIDs, 85% received MTX, 24% received SSZ, 54% received TNFi, and 8.1% received pamidronate. Compared with the aforementioned study, physicians in our cohort prescribed MTX more frequently and pamidronate less frequently. This tendency may also be associated with clinicians’ prior experience and familiarity with these drugs.

In CNO patients unresponsive to NSAIDs, conventional DMARDs such as MTX and SSZ are commonly used. Reported remission rates for MTX vary widely in the literature, ranging from 20% to 82% [7,19,20,21,22]. In our cohort, remission was achieved in 30.4% of patients treated with MTX and only 5.3% of those treated with SSZ. For patients with more active or refractory disease, TNF inhibitors are preferred based on their proven role in reducing TNF–α–mediated inflammation and bone damage [23]. Previous studies have reported remission rates between 46% and 89% within the first three months [13,14,18,19] with more recent data reporting improvement rates of up to 90.9% [19,20,24,25]. In our study, 93 patients received TNFi therapy, of whom 74.2% achieved clinical remission, which closely aligns with previously reported outcomes. Pamidronate has been shown to improve pain, physical function, and quality of life in CNO [26,27,28,29,30], with reported remission rates ranging from 69.4% to 91% [7,9,22,29,31,32,33]. It is often preferred in cases with spinal or mandibular involvement due to its potential to rapidly control inflammation and prevent structural damage [9,33]. In our cohort, pamidronate was used less frequently: 14 patients received a median of four monthly doses. Clinical remission was achieved in 5 patients (35.7%), and 8 patients showed a partial response.

The overlapping, sequential, and often combined use of csDMARDs and TNFi complicates the evaluation of the efficacy of individual treatments in CNO. Comparative data on TNFi and bisphosphonates are scarce; in our study, the limited use of pamidronate hindered direct comparisons. No significant differences were observed between the csDMARD and TNFi groups in terms of age at symptom onset, age at diagnosis, gender, or inflammatory markers (CRP, ESR). However, TNFi was more commonly prescribed in patients with active spinal lesions, longer disease duration, and more MRI lesions, suggesting a more aggressive disease phenotype. CDAS scores at diagnosis were similar in both groups (median 16 [IQR: 14–19] in the csDMARD group vs. 15.5 [IQR: 13–17.5] in the TNFi group), as were scores at the final visit (median 0 [IQR: 0–4] in the csDMARD group vs. 1 [IQR: 0–5] in the TNFi group), indicating comparable disease activity outcomes. Overall, clinicians in our country predominantly initiated treatment with the first CTP (csDMARDs), escalating to the second CTP (TNFi, with or without MTX) when needed, whereas the third CTP (bisphosphonates) was used infrequently. Few studies have evaluated clinical disease activity scoring in CNO. Before the development of standardized tools, disease activity was primarily assessed by medical history and physical examination (91%), inflammatory markers (84%), regional MRI (66%), and whole-body MRI (41%) [34]. Beck et al. [35] proposed the PedCNO score, combining ESR, CHAQ, MRI lesion count, and physician and patient global assessments; Zhao et al. [36] suggested an MRI-based grading system. In 2023, the CNO CDAS was introduced using CHOIR data to provide a standardized clinical tool [15]. In our study, the CNO CDAS proved both practical and feasible for assessing baseline status and treatment response. This multicenter study highlights the utility of the CNO CDAS as a practical tool for assessing clinical disease activity. Its consistency with treatment decisions and clinical findings supports its integration into routine care.

Beyond pharmacological treatment, supportive care is an important component of CNO management. Physical therapy and a gradual return to activity may help maintain strength and mobility, while individualized pain-management strategies and school or activity accommodations may support daily functioning. Surgery is uncommon but may be necessary for structural complications, such as fractures or spinal instability, rather than for treating inflammation itself. Because infection and malignancy may mimic CNO, careful diagnostic evaluation is essential, and antibiotics are not indicated unless a concomitant infection is present. New weakness, numbness, gait difficulties, or bowel or bladder dysfunction warrants urgent clinical assessment, particularly in patients with spinal involvement.

This study has several limitations. Its retrospective, multicenter design may have introduced variability in the timing and documentation of clinical assessments, despite inclusion being restricted to patients with complete data for the required study variables. The total numbers of patients screened and excluded at each center and the reasons for exclusion were unavailable, limiting assessment of potential selection bias. Restricting inclusion to patients with regular follow-up and complete data may also limit the generalizability of our findings. Differences across institutions in clinical decision-making, treatment selection, patient assessment practices, and radiologic interpretation could also have contributed to inter-center variability and systematic bias. Treatment selection may have been influenced by disease severity, prior treatment response, physician preferences, and center-specific practices, potentially introducing confounding by indication. Access to specialist care and medications was not systematically assessed, and its potential impact on treatment selection and outcomes could not be evaluated. The independent effects of follow-up duration, age, and concurrent arthritis on remission were not assessed in a multivariable analysis. Longer follow-up may have increased the opportunity to observe remission, potentially affecting comparisons of treatment outcomes. Moreover, the subjective nature of certain CDAS components may have increased inter-rater variability, influencing the interpretation of disease activity. The absence of standardized MRI protocols across centers further limits the comparability and consistency of imaging findings, potentially affecting the reliability of MRI-based assessments. The concordance between MRI lesions and clinically reported sites of pain was not systematically assessed across centers. Therefore, we could not evaluate the reliability of pain localization as an indicator of MRI-detected lesions. Furthermore, as follow-up MRI was not performed in all patients, the lower frequency of detected sacroiliitis at the final visit should be interpreted cautiously and does not establish radiological resolution in all initially affected patients.

## 5. Conclusions

In conclusion, our study found that TNF inhibitors (TNFi) were the most frequently used CTP regimen, especially in patients with spinal involvement or a greater number of MRI lesions and achieved the highest remission rate (40.4%) among 171 patients followed for a median of 36 months. The use of CTP-based analyses allowed for a standardized assessment of treatment strategies across different patient subgroups, facilitating better comparisons and clinical decision-making. These findings support the potential utility of the CNO CDAS for monitoring clinical disease activity and illustrate the value of CTPs as a framework for comparative effectiveness research. Prospective studies are needed to further validate the CNO CDAS and compare outcomes across CTP strategies.

## Figures and Tables

**Table 1:** Clinical Symptoms and Laboratory Parameters at Diagnosis.

	At Diagnosis (n = 171)	Treatment Modification Group (n = 89)	At Last Visit (n = 171)
Clinical Symptoms			
Bone pain, n (%)	163 (95.3%)	54 (60.7%)	13 (7.6%)
Impaired daily activity, n (%)	116 (67.8%)	43 (48.3%)	7 (4.1%)
Fatigue, n (%)	60 (35.1%)	21 (23.6%)	6 (3.5%)
Enthesitis, n (%)	19 (11.1%)	9 (10.1%)	5 (2.9%)
Bone deformity, n (%)	15 (8.8%)	3 (3.4%)	7 (4.1%)
Skin involvement, n (%)	9 (5.3%)	3 (3.4%)	2 (1.2%)
Gastrointestinal symptoms, n (%)	5 (2.9%)	2 (2.2%)	4 (2.3%)
Laboratory Parameters			
WBC (/mm^3^), median (IQR)	8360 (5560–11,140)	9100 (7280–12,500)	7600 (5728–10,072)
Hemoglobin (g/dL), median (IQR)	12.2 (11.25–13.95)	12.0 (10.4–13.6)	12.8 (11.13–14.47)
Platelets (/mm^3^), median (IQR)	365,000 (228,900–501,100)	364,000 (235,900–492,100)	309,500 (170,750–449,250)
ESR (mm/h), median (IQR)	25 (9–57)	32 (19–57)	9.5 (3–21.5)
CRP (mg/L), median (IQR)	3.0 (0–10)	5.0 (0–18)	0.7 (0–2.6)
LDH (U/L), median (IQR)	208 (172–284)	197 (175–220)	180 (151–236)
ALP (U/L), median (IQR)	174 (121–281)	143 (109–210)	168 (115–268)
Calcium (mg/dL), median (IQR)	9.6 (8.6–10.6)	9.4 (8.4–10.4)	9.8 (9.3–10.3)
Phosphate (mg/dL), median (IQR)	4.6 (3.6–5.6)	4.0 (2.45–5.55)	4.3 (3.25–5.35)

ALP, alkaline phosphatase; CRP, C-reactive protein; ESR, erythrocyte sedimentation rate; IQR, interquartile range; LDH, lactate dehydrogenase; WBC, white blood cell count.

**Table 2:** Distribution and Clinical Outcomes of Treatments in Patients Diagnosed with Chronic Nonbacterial Osteomyelitis.

Treatment	Patient Count n (%)	Remission n (%)	Partial Response n (%)	No Response n (%)	Duration (Months, IQR)
NSAIDs	171 (100)	-	88 (51.5)	83 (48.5)	1.5 (0.25–2.75)
Methotrexate	146 (85.4)	52 (30.4)	82 (48.0)	12 (7.0)	12 (7–22)
Sulfasalazine	41 (24.0)	9 (5.3)	10 (5.8)	22 (12.9)	6 (2–10.25)
TNFi	93 (54.4)	69 (40.4)	22 (12.9)	2 (1.2)	14 (0–43.5)
Pamidronate	14 (8.2)	5 (2.9)	8 (4.7)	1 (0.6)	4 doses

NSAIDs, non-steroidal anti-inflammatory drugs; TNFi, tumor necrosis factor inhibitors.

**Table 3:** Clinical Assessment Scores at Diagnosis, at Treatment Modification, and at the Last Visit in Patients with CNO.

Parameter	At Diagnosis	At Treatment Modification	At Last Visit	*p*-Value
CNO CDAS *	15 (13–18)	15 (13–18)	0 (0–5)	<0.001
• Number of clinically active lesions *	2 (1–3)	2 (1–3)	0 (0–0)	
• Patient pain assessment (0–10 scale) *	7 (6–9)	6 (5–7.5)	0 (0–2)	
• Global assessment score (0–10 scale) *	7 (6–9)	6 (5–7)	0 (0–2)	

CNO CDAS, Chronic Nonbacterial Osteomyelitis Clinical Disease Activity Score. * Parameters were given as median (IQR). The *p*-value refers to the overall comparison of CNO-CDAS scores at diagnosis, at treatment modification, and at the last visit using the Friedman test.

**Table 4:** Comparison of patients using only csDMARDs with patients who switched to TNFi.

Variables	csDMARD (n = 82)	TNFi (n = 83)	*p* Value
Age at symptom onset, years	10 (8–12)	10 (7–12.85)	0.728
Age at diagnosis, years	11 (9.25–12.75)	11 (7.65–13.7)	0.938
Female/Male, n	36/46	31/52	0.391
Disease duration, months	24 (9.25–38.75)	36 (22.65–50.7)	0.002
Clinical CNO lesion count	2 (1–3)	2 (1–3)	0.725
Total lesion count on MRI	4 (0.5–7.5)	10 (5–15)	0.001
Active spinal lesion, n (%)	11 (13.4%)	23 (27.7%)	0.020
CNO CDAS at diagnosis	16 (14–19)	15.5 (13–17)	0.266
CNO CDAS at last visit	0 (0–4)	1 (0–5)	0.288
CRP (mg/L)	2 (0.55–4.45)	3.8 (0.95–8.7)	0.053
ESR (mm/h)	23.5 (8.5–53.5)	31 (13–46)	0.199

CNO CDAS, Chronic Nonbacterial Osteomyelitis Clinical Disease Activity Score; csDMARD, conventional synthetic Disease-Modifying Antirheumatic Drug; CRP, C-reactive protein; ESR, erythrocyte sedimentation rate; MRI, magnetic resonance imaging; TNFi, tumor necrosis factor inhibitor.

**Table 5:** Comparison of CNO CDAS Scores Across Treatment Groups.

	csDMARD or NSAIDs to TNFi (n = 84)	TNFi to Another TNFi (n = 2)	csDMARD to Pamidronate (n = 2)	TNFi to Pamidronate (n = 1)	*p* Value
CNO CDAS at diagnosis, median (IQR)	15 (9–27)	13.5 (13–14)	15.5 (15–16)	16	0.805
CNO CDAS at the time of the treatment change, median (IQR)	15 (4–26)	13.5 (13–14)	17 (16–18)	15	0.616
CNO CDAS at last visit, median (IQR)	1 (0–20)	2.5 (0–5)	6 (5–7)	7	0.342

CNO CDAS, Chronic Nonbacterial Osteomyelitis Clinical Disease Activity Score; csDMARD, conventional synthetic Disease-Modifying Antirheumatic Drug; NSAIDs, non-steroidal anti-inflammatory drugs; TNFi, tumor necrosis factor inhibitor. Each *p*-value refers to the comparison of CNO-CDAS scores among the four treatment transition groups at the corresponding assessment time point.

## Data Availability

The datasets used and analyzed during the current study are available from the corresponding author upon reasonable request. Restrictions may apply due to patient confidentiality and ethical guidelines.

## Abbreviations

BMD	Bone mineral density
CARRA	Childhood Arthritis and Rheumatology Research Alliance
CDAS	Clinical Disease Activity Score
CHAQ	Childhood Health Assessment Questionnaire
CHOIR	Chronic Nonbacterial Osteomyelitis International Registry
CNO	Chronic nonbacterial osteomyelitis
CRP	C-reactive protein
csDMARDs	Conventional synthetic disease-modifying antirheumatic drugs
CTPs	Consensus treatment plans
DMARDs	Disease-modifying antirheumatic drugs
ESR	Erythrocyte sedimentation rate
IQR	Interquartile range
MRI	Magnetic resonance imaging
MTX	Methotrexate
NSAIDs	Non-steroidal anti-inflammatory drugs
PeRA	Pediatric Rheumatology Academy
PET-CT	Positron emission tomography-computed tomography
SPSS	Statistical Package for the Social Sciences
STIR	Short tau inversion recovery
TNF	Tumor necrosis factor
TNFi	Tumor necrosis factor inhibitors
SSZ	Sulfasalazine
